# A facile method for the preparation of bifunctional Mn:ZnS/ZnS/Fe_3_O_4_ magnetic and fluorescent nanocrystals

**DOI:** 10.3762/bjnano.6.178

**Published:** 2015-08-17

**Authors:** Houcine Labiadh, Tahar Ben Chaabane, Romain Sibille, Lavinia Balan, Raphaël Schneider

**Affiliations:** 1 Unité de Recherche Synthèse et Structure de Nanomatériaux UR 11 ES 30, Université de Carthage, Faculté des Sciences de Bizerte, 7021 Jarzouna, Bizerte, Tunisia; 2 Institut Jean Lamour (IJL), Université de Lorraine, CNRS, UMR 7198, CNRS, BP 70239, 54506 Vandoeuvre-lès-Nancy Cedex, France; 3 Institut de Science des Matériaux de Mulhouse (IS2M), CNRS, UMR 7361, 15 rue Jean Starcky, 68093 Mulhouse, France,; 4 Université de Lorraine, Laboratoire Réactions et Génie des Procédés (LRGP), UMR 7274, CNRS, 1 rue Grandville, BP 20451, 54001 Nancy Cedex, France

**Keywords:** aqueous synthesis, bifunctional nanocrystals, magnetite, Mn-doped ZnS, quantum dots

## Abstract

Bifunctional magnetic and fluorescent core/shell/shell Mn:ZnS/ZnS/Fe_3_O_4_ nanocrystals were synthesized in a basic aqueous solution using 3-mercaptopropionic acid (MPA) as a capping ligand. The structural and optical properties of the heterostructures were characterized by X-ray diffraction (XRD), dynamic light scattering (DLS), transmission electron microscopy (TEM), UV–vis spectroscopy and photoluminescence (PL) spectroscopy. The PL spectra of Mn:ZnS/ZnS/Fe_3_O_4_ quantum dots (QDs) showed marked visible emission around 584 nm related to the ^4^T_1_ → ^6^A_1_ Mn^2+^ transition. The PL quantum yield (QY) and the remnant magnetization can be regulated by varying the thickness of the magnetic shell. The results showed that an increase in the thickness of the Fe_3_O_4_ magnetite layer around the Mn:ZnS/ZnS core reduced the PL QY but improved the magnetic properties of the composites. Nevertheless, a good compromise was achieved in order to maintain the dual modality of the nanocrystals, which may be promising candidates for various biological applications.

## Introduction

Semiconductor nanocrystals with a diameter of approximately 1–10 nm, also referred to as quantum dots (QDs), have attracted great attention due to their unique optical and electronic properties, which are not observed in bulk semiconductor materials [1–3]. Compared to conventional organic dyes, QDs possess many advantages, including a broad absorption with a narrow photoluminescence (PL) spectra, low photobleaching, high PL quantum yield (QY), tunable emission from the visible to infrared wavelengths, and high resistance to chemical degradation [3–4]. Such characteristics originate from their large surface-to-volume ratio and from confinement phenomena resulting in an atomic-like electronic structure with discrete energy levels. Thus, QDs have been widely studied for their fundamental properties and applications, mostly employed as emitters for biolabelling [3,5], light emitting diodes [6–7] or solar cells [8]. Conventional QDs systems have a core/shell architecture. The shell, generally constituted of a wide band gap material such as ZnS, prevents degradation and preserves the optical properties [3–4].

Magnetic nanoparticles have many advantages, especially for biological applications. They can, for example, be used as a contrast agent in magnetic resonance imaging or as therapeutic agents in magnetic fluid hyperthermia [9–10]. Several successful applications such as targeted drug delivery, bioseparation, biodetection, and labelling and sorting of cells [11–14] are based on the unique feature of magnetic nanoparticles to respond well to magnetic control.

The combination of magnetic and optical properties in a single, nanostructured material has attracted increased attention because of the advantageous properties of such nanoparticles. Such fluorescent and magnetic properties allow nanoparticles to be guided with a magnetic force both in vivo and in vitro and provide detailed information on their biodistribution using a fluorescence microscope [15–17]. Such bifunctional nanoparticles would enable simultaneous biolabelling/imaging and cell sorting/separation. Over the last decades, different strategies have been developed toward this goal such as epitaxial heterocrystalline growth, co-encapsulation of preformed QDs and magnetic particles in silica beads, doping of QDs with transition metal ions, conjugation between magnetic chelates or magnetic nanocrystals with QDs (e.g., using avidin and biotin) [18–19]. However, there are still some challenges to overcome such as the complexity in the preparation, which involves multistep synthesis and purification stages or the instability and aggregation of the nanocomposites in aqueous solution. Moreover, some of the nanocomposite particles are rather large (70–200 nm), limiting their use in biological applications.

Epitaxial heterocrystalline growth is generally conducted by coating a ferro- or ferri-magnet that has an ordering temperature well above 300 K (e.g., FePt, Fe_2_O_3_, or Fe_3_O_4_) with a semiconductor shell (CdSe or CdS) of thickness between 2–7 nm resulting in either spherical core/shell nanoparticles or heterodimers [20–22]. The PL QY of the resulting bifunctional nanoparticles is generally low (typically <5%) due to the quenching effect of the magnetic domain [20–21].

Herein, we describe a facile synthesis of epitaxial heterocrystalline nanoparticles, consisting of an Fe_3_O_4_ shell surrounding a core/shell of Mn-doped ZnS/ZnS QDs. The choice of the Mn:ZnS/ZnS system was motivated by its very low toxicity and thus potential use in various biological applications such as fluorescent labelling [5,23–24]. Mn:ZnS/ZnS QDs were first prepared in aqueous solution in the presence of the MPA ligand. The Fe_3_O_4_ layer was next grown on the preformed Mn:ZnS/ZnS QDs in aqueous solution. The resulting bifunctional, core/shell/shell Mn:ZnS/ZnS/Fe_3_O_4_ QDs exhibited superparamagnetism and fluorescence properties, which are discussed herein. The approach described in this study is anticipated to be useful and cost-effective for biological and biomedical applications requiring both fluorescence and magnetic characteristics.

## Results and Discussion

### Synthesis and structural/microstructural characterization

**Figure 1:**
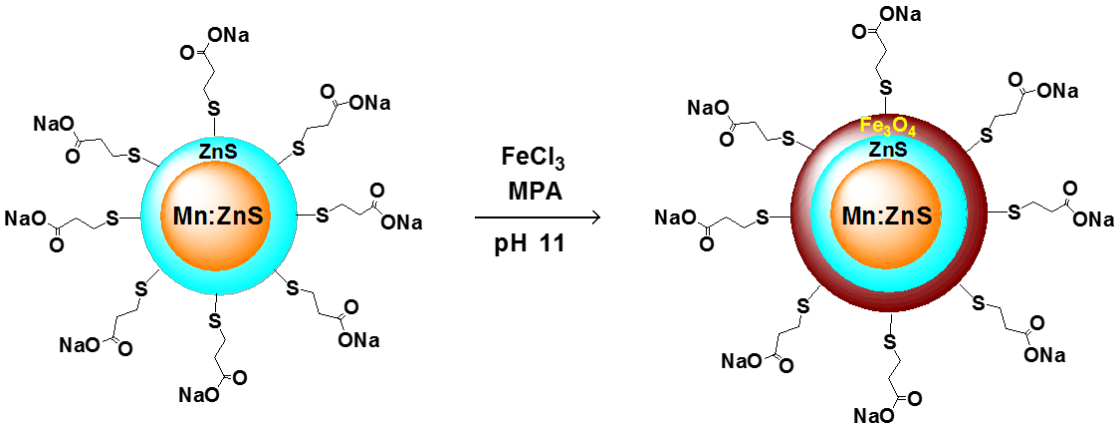
Schematic of the core/shell/shell Mn:ZnS/ZnS/Fe_3_O_4_ QD synthesis.

The design of the fluorescent and magnetic Mn:ZnS/ZnS/Fe_3_O_4_ QDs is shown schematically in Figure 1. The Fe_3_O_4_ shell is grown in aqueous solution on the surface of MPA-capped core/shell Mn:ZnS/ZnS nanocrystals prepared via the nucleation-doping strategy [5,23–25]. Various amounts of a 0.1 M FeCl_3_ aqueous solution and of the MPA ligand (1, 1.5, 2 or 3 mL) were injected dropwise at 100 °C to the preformed Mn:ZnS/ZnS QDs dispersion solution to form magnetic Fe_3_O_4_ layers of different thicknesses. These Mn:ZnS/ZnS/Fe_3_O_4_ samples are respectively labelled (1), (1.5), (2) and (3) hereafter.

**Figure 2:**
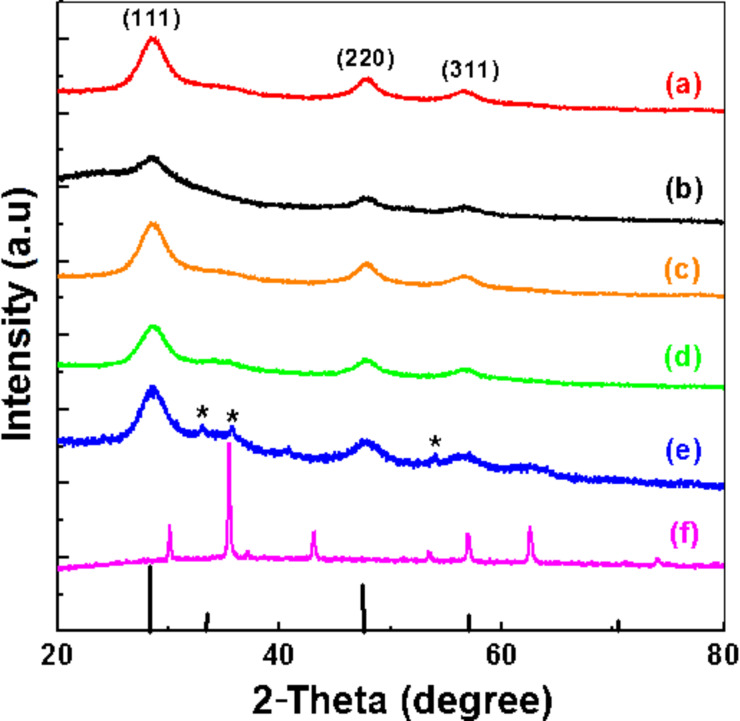
XRD patterns of (a) Mn:ZnS/ZnS, (b–e) Mn:ZnS/ZnS/Fe_3_O_4_ (1), (1.5), (2) and (3) samples, respectively and (f) Fe_3_O_4_ nanocrystals. The reference line spectrum (bottom) shows the relative intensities of the reflections for bulk ZnS. The diffraction peaks of hematite (α-Fe_2_O_3_) in the XRD pattern f are labelled with an asterisk.

X-ray diffraction (XRD) patterns of Mn:ZnS/ZnS, Mn:ZnS/ZnS/Fe_3_O_4_ and Fe_3_O_4_ nanocrystals are shown in Figure 2. All the diffraction peak positions of the Fe_3_O_4_ black powder prepared in the absence of Mn:ZnS/ZnS QDs are in accordance with the data reported in the literature (see JCPDS record number 99-100-7775) and correspond to the magnetite Fe_3_O_4_ spinel-like structure (Figure 2f) [26–27]. Note that this sample was prepared using FeCl_3_ as a precursor in the presence of the MPA ligand, which probably also acts as a reducing agent to provide the necessary amount of Fe^2+^ to form magnetite. For the Mn:ZnS/ZnS and Mn:ZnS/ZnS/Fe_3_O_4_ (1), (1.5), (2) and (3) nanocrystals, the diffraction peaks match perfectly with the (111), (220), and (311) crystalline planes of the cubic ZnS phase (JCPDS record number 99-100-0108) [26]. The XRD patterns of Mn:ZnS/ZnS/Fe_3_O_4_ QDs (2) and (3) exhibit additional, low intensity peaks that were not observed in the case of Mn:ZnS/ZnS/Fe_3_O_4_ (1) and (1.5) nanocrystals. These peaks correspond to magnetite and were detectable only when the thickness of the magnetic shell increased. For the Mn:ZnS/ZnS/Fe_3_O_4_ (3) sample, the appearance of additional peaks of very low intensity originating from hematite α-Fe_2_O_3_ (JCPDS record number 99-100-0140) can also be observed [26]. Since the surface of finely divided materials is highly reactive, partial oxidation of Fe_3_O_4_ into Fe_2_O_3_ may have taken place during the handling of the nanocrystals [28–29]. The crystallites sizes of the Mn:ZnS/ZnS/Fe_3_O_4_ nanoparticles were calculated using the Scherrer formula based on the width of the most intense (111) diffraction peak (Table 1).

**Table 1:** Calculated crystallite diameters and measured hydrodynamic diameters.

Sample	Diameter (nm)^a^	Hydrodynamic diameter (nm)^b^

Mn:ZnS/ZnS	3.5 ± 1.0	11 ± 1.0
Mn:ZnS/ZnS/Fe_3_O_4_ (1)	4.2 ± 1.0	22 ± 4.7
Mn:ZnS/ZnS/Fe_3_O_4_ (1.5)	3.9 ± 1.0	23 ± 2.9
Mn:ZnS/ZnS/Fe_3_O_4_ (2)	4.0 ± 1.0	24 ± 7.5
Mn:ZnS/ZnS/Fe_3_O_4_ (3)	4.5 ± 1.0	39 ± 8.0

^a^Determined using the Scherrer equation; ^b^Determined by DLS measurements.

**Figure 3:**
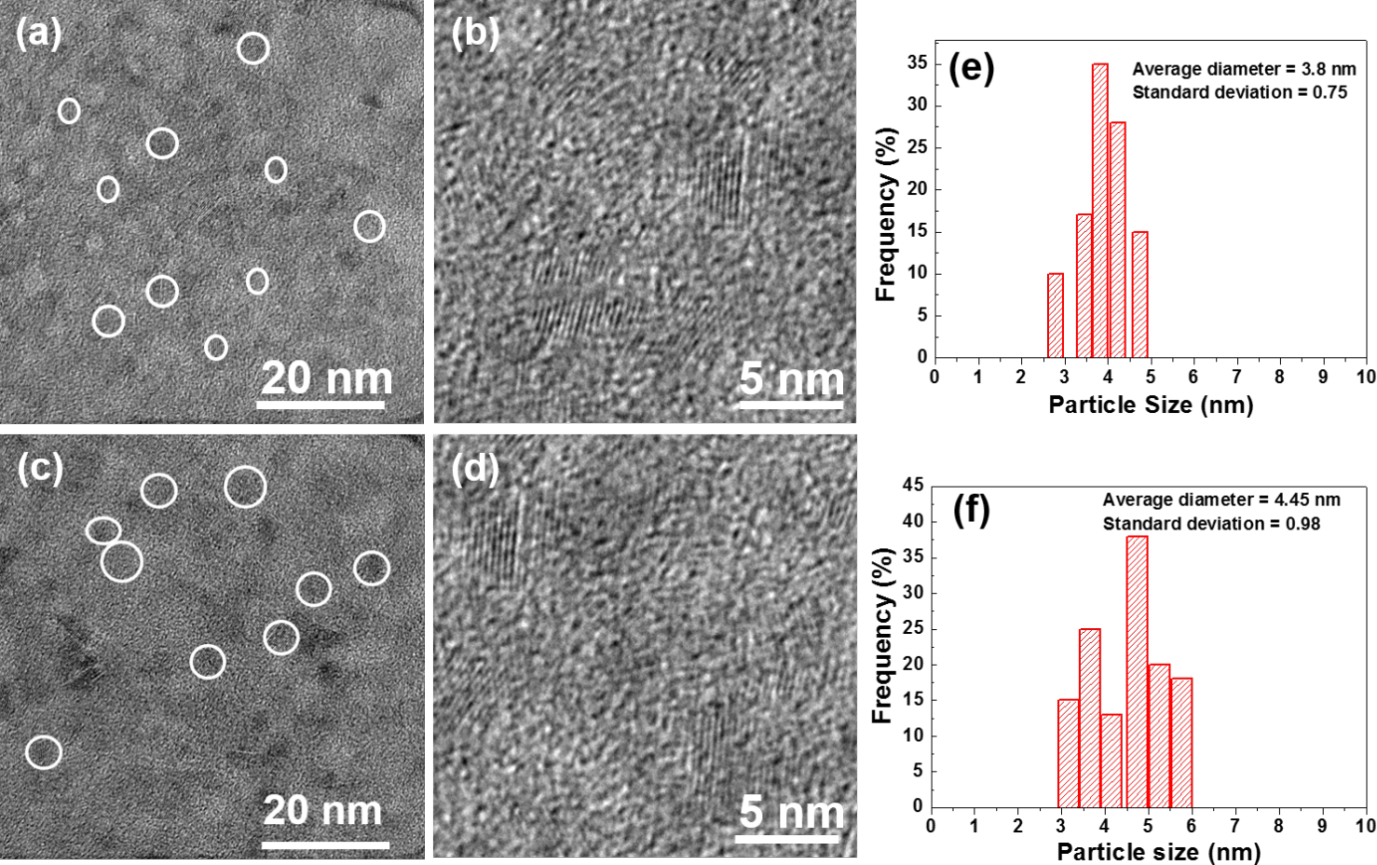
TEM and HR-TEM micrographs of (a) Mn:ZnS/ZnS and (b) Mn:ZnS/ZnS/Fe_3_O_4_ (1.5) nanocrystals, and (c) and (d) are the corresponding size distributions.

Figure 3 shows transmission electron microscopy (TEM) and high-resolution TEM (HR-TEM) images of the Mn:ZnS/ZnS and Mn:ZnS/ZnS/Fe_3_O_4_ (1.5) materials. All Mn:ZnS/ZnS core/shell nanocrystals were nearly monodisperse and of spherical shape. The average diameter of these QDs was determined to be 3.8 ± 0.7 nm (Figure 3a,b,e). The TEM and HR-TEM micrograph of Mn:ZnS/ZnS/Fe_3_O_4_ (1.5) crystals shows almost spherical particles with an average size of 4.4 ± 0.9 nm (Figure 3c,d,f). The measured diameters fit well with the calculated crystallite sizes deduced from the application of the Scherrer formula (Table 1). The increase in size observed from 3.8 ± 0.7 nm to 4.4 ± 0.9 nm confirms the growth of the iron oxide shell around the Mn:ZnS/ZnS core and indicates that the thickness of the Fe_3_O_4_ layer is approximately 0.6 nm for Mn:ZnS/ZnS/Fe_3_O_4_ (1.5) QDs.

**Figure 4:**
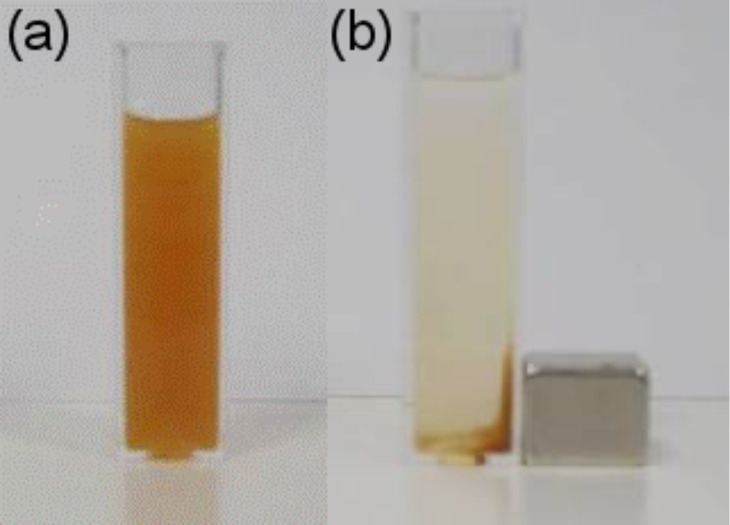
Colloidal dispersion of Mn:ZnS/ZnS/Fe_3_O_4_ (2) nanoparticles dispersed in water (a) in the absence of and (b) in the presence of a permanent magnet.

The nanoparticles were characterized by their hydrodynamic diameter before and after the Fe_3_O_4_ coating using dynamic light scattering (DLS) (Table 1). The average hydrodynamic diameters, *d*_H_, are larger than the particles sizes determined using TEM because of the solvation layer around the QDs in aqueous solution. The lowest *d*_H_ value of 11 nm was obtained for the Mn:ZnS/ZnS QDs. The *d*_H_ increased with the thickness of the magnetic shell (Table 1). The highest *d*_H_ (39 nm) was observed for Mn:ZnS/ZnS/Fe_3_O_4_ (3) nanocrystals. The DLS measurements clearly confirmed the growth of the Fe_3_O_4_ shell around the Mn:ZnS/ZnS core. Note also that these functionalized nanoparticles could be easily redispersed in aqueous medium after purification and drying (Figure 4a). The room temperature, magnetic harvesting of the heterostructured nanocrystals could be conducted for all Mn:ZnS/ZnS/Fe_3_O_4_ materials prepared. Figure 4b illustrates the sensitive magnetic response of the Mn:ZnS/ZnS/Fe_3_O_4_ (2) sample.

### PL properties of Mn:ZnS/ZnS/Fe_3_O_4_ QDs

**Figure 5:**
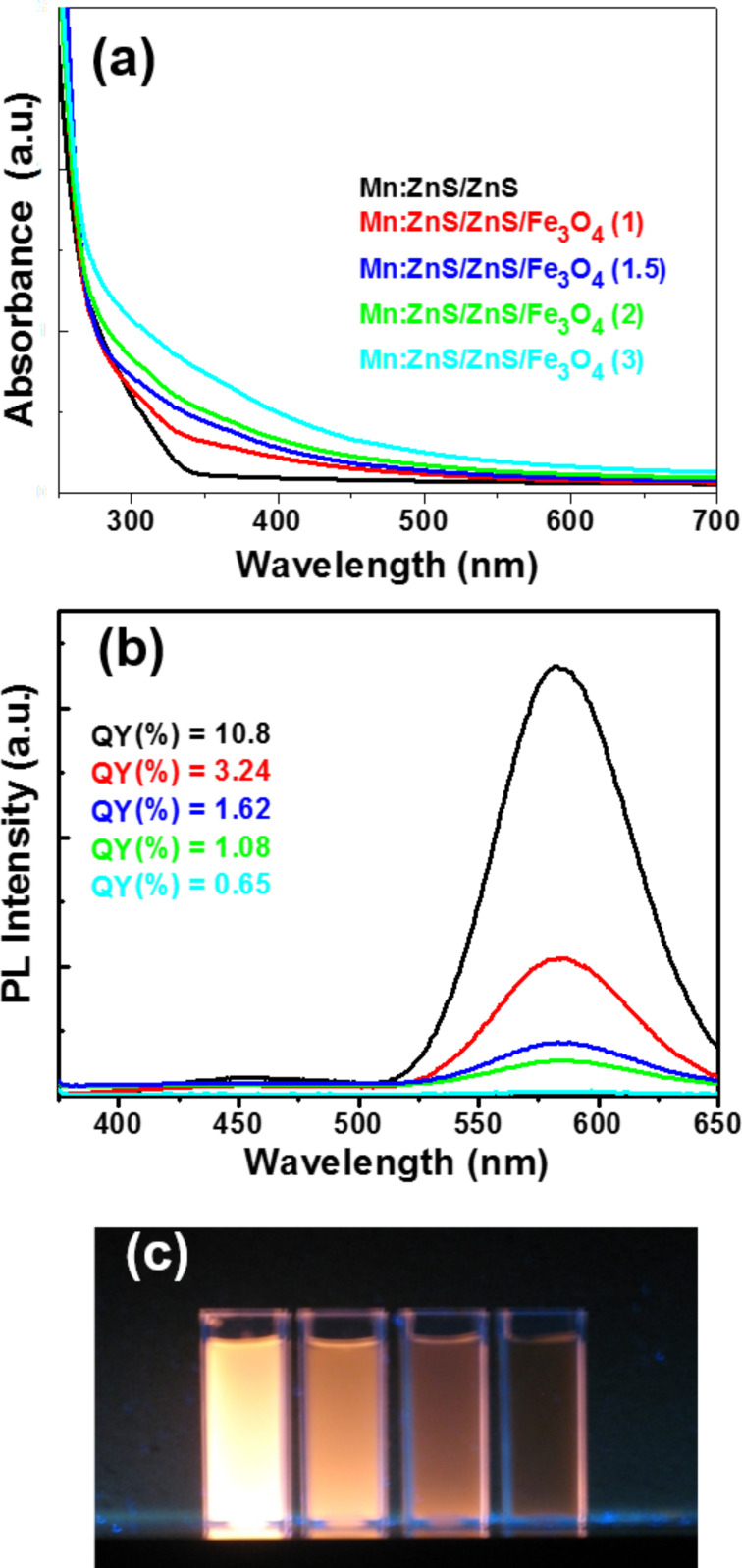
(a) Absorption and (b) photoluminescence spectra of Mn:ZnS/ZnS and Mn:ZnS/ZnS/Fe_3_O_4_ (1), (1.5), (2) and (3) nanocrystals. (c) Digital photograph of colloidal dispersions of Mn:ZnS/ZnS/Fe_3_O_4_ nanocrystals with increasing Fe_3_O_4_ shell thickness.

The optical properties of Mn:ZnS/ZnS/Fe_3_O_4_ samples were studied by UV–vis absorption spectroscopy and PL spectroscopy (Figure 5a,b). As can be seen, the absorbance of Mn:ZnS/ZnS/Fe_3_O_4_ crystals (Figure 5a) shifted to longer wavelengths with increasing thickness of the magnetic layer. The PL spectrum of Mn:ZnS/ZnS/Fe_3_O_4_ nanocrystals exhibits two bands: a broad and very weak one centered at approximately 450 nm and another dominant one at approximately 584 nm (visible, orange wavelength region). The first emission is associated with transitions involving vacancy states of the ZnS host material [24,30], while the second one originates from the Mn^2+^ dopant, which is excited via energy transfer of the ZnS host followed by the dipole forbidden ^4^T_1_ → ^6^A_1_ ligand field transition [23–24,31].

Adding the magnetic Fe_3_O_4_ shell to the surface of Mn:ZnS/ZnS QDs progressively reduced the fluorescence intensity, which became very low especially for the Mn:ZnS/ZnS/Fe_3_O_4_ (3) material (Figure 5b,c). Note that the PL QY decreased from 10.8% for the Mn:ZnS/ZnS QDs, to 3.24% for the Mn:ZnS/ZnS/Fe_3_O_4_ (1) material. A continuous decrease of the PL QY was further observed when increasing the thickness of the Fe_3_O_4_ shell and reached 0.65% for Mn:ZnS/ZnS/Fe_3_O_4_ (3). These results probably originate (1) from structural defects related to the notable discrepancy between the lattice parameters of the cubic zinc blende phase of the ZnS core material (*a* = 5.4093 Å) and the cubic spinel phase of the magnetite shell (*a* = 8.3970 Å) and (2) from the partial quenching of the Fe_3_O_4_ paramagnetic shell originating from the changes in the electronic density on the surface of Mn:ZnS/ZnS QDs.

### Magnetic properties of Mn:ZnS/ZnS/Fe_3_O_4_ nanocrystals

**Figure 6:**
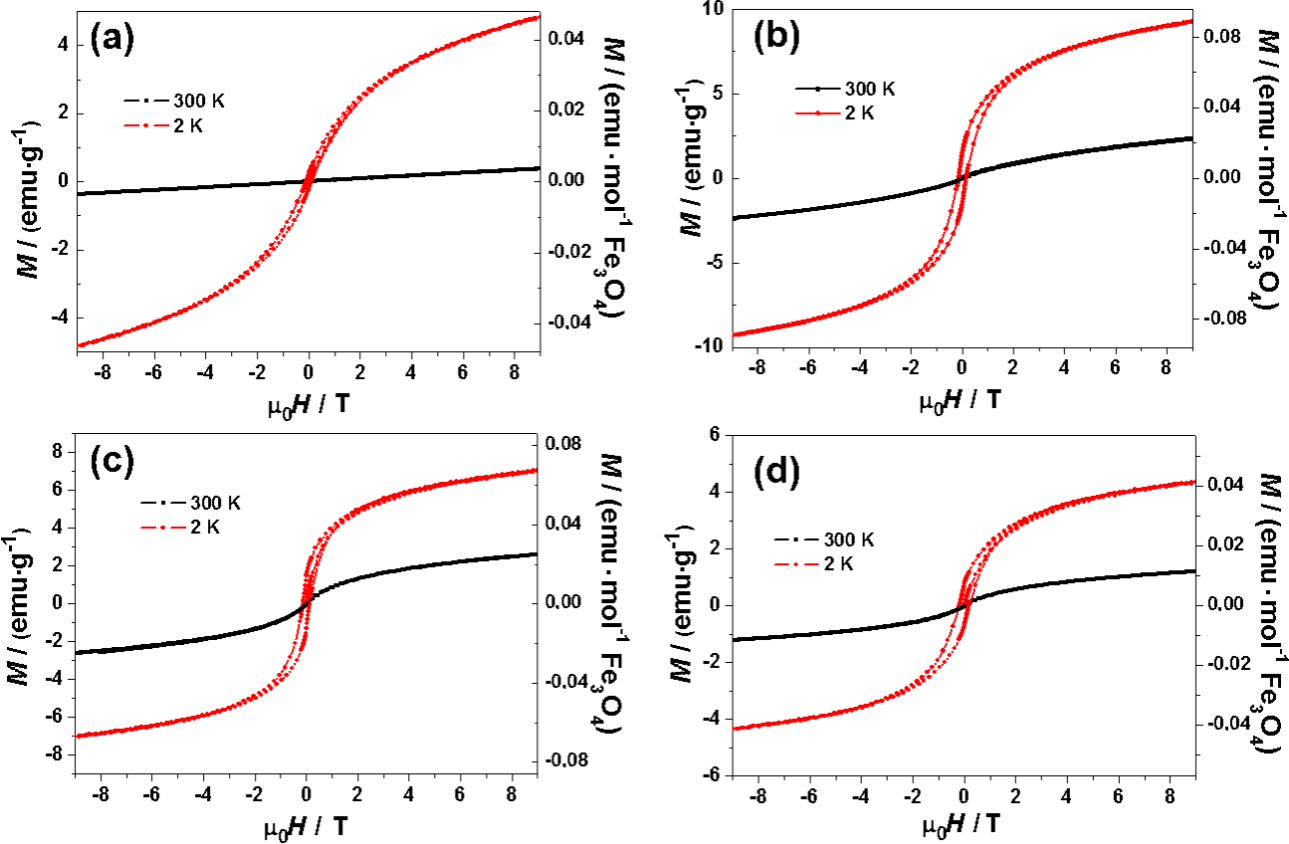
(a–d) Magnetization loops of fluorescent, magnetic nanocrystals Mn:ZnS/ZnS/Fe_3_O_4_ (1, 1.5, 2 and 3). The units of magnetization are given in emu/g of the total weight, and in emu/mole of Fe_3_O_4_.

The magnetization curves, *M* versus μ_0_*H*, of Mn:ZnS/ZnS/Fe_3_O_4_ nanocrystals at 300 K and 2 K are shown in Figure 6. At 300 K, a strong paramagnetic signal was observed for the Mn:ZnS/ZnS/Fe_3_O_4_ samples (1.5), (2) and (3). The magnetization values recorded at the maximum applied magnetic field of 9 T, *M*_9T_, are given in Table 2.

**Table 2:** Magnetic characteristics of Mn:ZnS/ZnS/Fe_3_O_4_ nanocrystals.

Sample	*M*_9T_ (2 K, 300 K) (emu/g)	*M*_R_ (2K) (emu/g)	Coercive field (T)	*T*_B_ (K)

Mn:ZnS/ZnS/Fe_3_O_4_ (1)	4.82, —	0.21	0.08	6
Mn:ZnS/ZnS/Fe_3_O_4_ (1.5)	9.30, 2.33	1.45	0.13	12
Mn:ZnS/ZnS/Fe_3_O_4_ (2)	7.05, 2.67	1.61	0.15	20
Mn:ZnS/ZnS/Fe_3_O_4_ (3)	4.35, 1.21	0.77	0.18	21

The highest value of 2.67 emu/g was obtained for the sample Mn:ZnS/ZnS/Fe_3_O_4_ (2). This value was remarkably lower than the saturation magnetization of bulk phase Fe_3_O_4_ (90 emu/g), which could be related to the quantum confinement effects of nanocrystals and to the diamagnetic contribution of the ZnS core.

At 2 K, all bifunctional nanoparticles exhibited hysteresis with remnance magnetization, *M*_R_, at 9 T and coercivity, H_C_, indicating a dominant ferromagnetic nature of the iron oxide layer. The magnetic characteristics (*M*_R_, *M*_9T_, and *H*_C_) of the samples are given in Table 2. One can observe that the hysteresis loops are not saturated even for fields up to ±9 T; this could be due to frozen spins at the surface of the nanocrystals as reported in previous works [32–33]. The coercive fields increased with the iron oxide layer thickness, where the highest *H*_C_ value of 0.18 T was measured for Mn:ZnS/ZnS/Fe_3_O_4_ QDs (3). Thus, the nanocrystals become magnetically harder with an increasingly open hysteresis loop with increasing thickness of the magnetite shell.

The values of *M*_R_ and *M*_9T_ change as well in the same manner for Mn:ZnS/ZnS/Fe_3_O_4_ QDs (1, 1.5 and 2). Surprisingly, the Mn:ZnS/ZnS/Fe_3_O_4_ (3) crystals exhibited reduced *M*_R_ and *M*_9T_ values, which were lower than those measured for (1.5) and (2) samples. However, its coercive field was the highest (Table 2). This likely originates from the contribution of the hematite (α-Fe_2_O_3_) impurity in the Mn:ZnS/ZnS/Fe_3_O_4_ (3) sample, as observed in the XRD pattern. Among bulk iron oxides (Fe_3_O_4_, α-Fe_2_O_3_ and γ-Fe_2_O_3_), hematite exhibits the lowest saturation magnetization of 0.3 emu/g with a relatively large coercivity of 0.17 T at room temperature [34–35].

**Figure 7:**
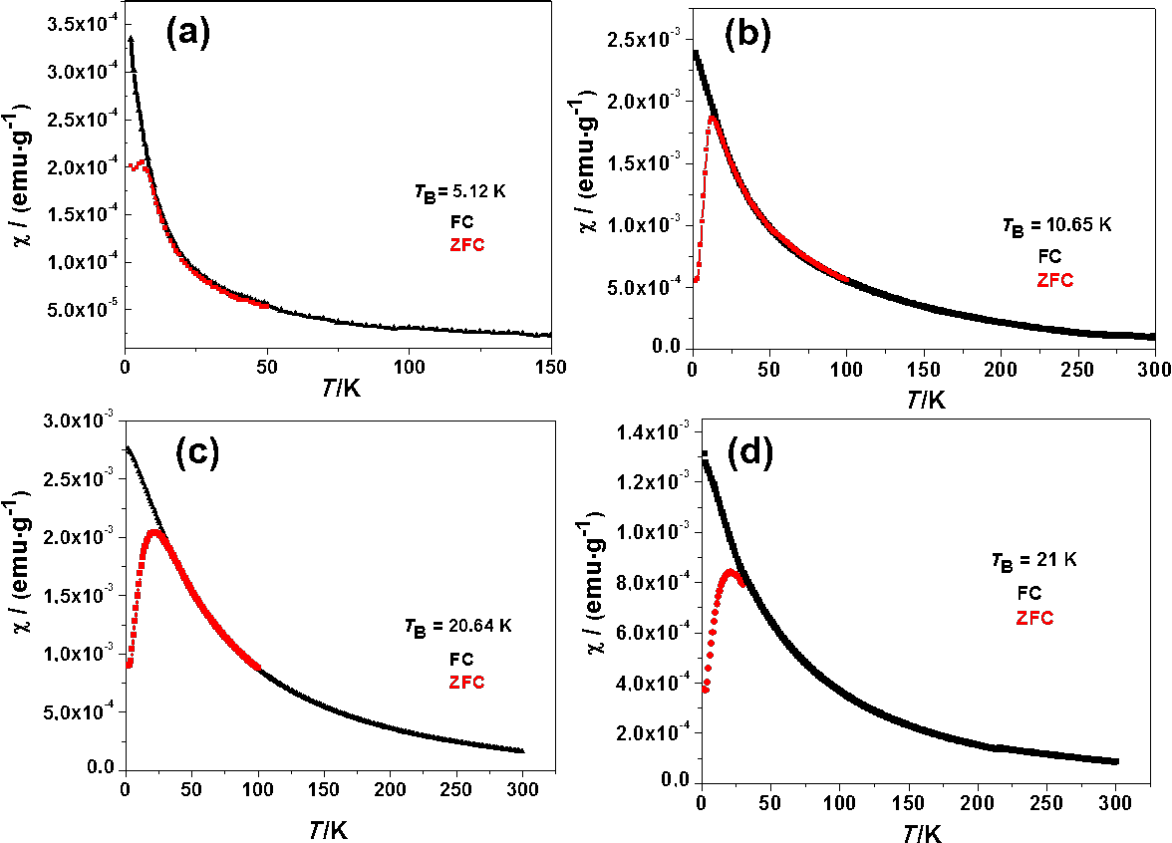
FC and ZFC thermal variation of the susceptibility of the samples measured in a magnetic field of 500 Oe: (a) Mn:ZnS/ZnS/Fe_3_O_4_ (1), (b) Mn:ZnS/ZnS/Fe_3_O_4_ (1.5), Mn:ZnS/ZnS/Fe_3_O_4_ (c) (2) and (d) Mn:ZnS/ZnS/Fe_3_O_4_ (3). The blocking temperature, *T*_B_, is indicated in each graph.

The zero-field-cooled (ZFC) and field-cooled (FC) measurements were performed under an applied field of 500 Oe between 2–300 K. The ZFC and FC curves of Mn:ZnS/ZnS/Fe_3_O_4_ (1, 1.5 and 2) crystals are given in Figure 7. The ZFC curve reached a temperature maximum corresponding to the blocking temperature (*T*_B_) of the sample. The result of the ZFC and FC measurements confirmed the superparamagnetic behavior of the nanocrystals [36]. Below the blocking temperature, the material is ferromagnetic, and above *T*_B_, it is superparamagnetic [33].

The values for the *T*_B_ are given in Table 2. *T*_B_ increased with increased iron oxide shell thickness starting at 6 K for Mn:ZnS/ZnS/Fe_3_O_4_ (1) at 20 K for Mn:ZnS/ZnS/Fe_3_O_4_ (2). These results are in agreement with the size increase of the nanocrystals [36].

## Conclusion

In summary, we have developed a low cost and efficient aqueous-based route to prepare core/shell/shell Mn:ZnS/ZnS/Fe_3_O_4_ nanocrystals possessing both fluorescent and magnetic properties. Our results showed that increasing the thickness of the iron oxide shell around the Mn:ZnS/ZnS QDs improved the magnetic properties of the heterostructured nanomaterials but negatively affected their luminescence efficiency, and vice versa. The Mn:ZnS/ZnS/Fe_3_O_4_ (1) sample, which exhibited the best fluorescence quantum yield, also exhibited limited magnetic characteristics. Nevertheless, samples Mn:ZnS/ZnS/Fe_3_O_4_ (1.5) and Mn:ZnS/ZnS/Fe_3_O_4_ (2) are a good compromise to maintain both fluorescence and magnetic properties. We therefore believe that the synthetic protocol developed in this work may pave a reliable way for constructing imaging probes with good performance and low toxicity for biological applications.

## Experimental

### Chemicals

Zinc sulfate heptahydrate (ZnSO_4_·7H_2_O, 99.99%), manganese acetate tetrahydrate (Mn(OAc)_2_·4H_2_O, 99%), iron(III) chloride hexahydrate (FeCl_3_·6H_2_O, 99%), sodium sulfide nonahydrate (Na_2_S·9H_2_O, 98+%), 3-mercaptopropionic acid (MPA, 99%) and isopropanol (HPLC grade) were used as received without additional purification. All solutions were prepared using Milli-Q^®^ water (18.2 MΩ·cm, Millipore Corporation) as the solvent.

### Synthesis of Mn:ZnS/ZnS QDs

The Mn:ZnS/ZnS nanoparticles were prepared in basic aqueous solution at 100 °C using MPA as a ligand. The synthesis was carried out according to the procedure detailed in our previous work [37]. The obtained colloidal solution of Mn:ZnS/ZnS QDs was labelled as solution A. The Mn:ZnS/ZnS QDs were precipitated by adding isopropanol, collected by centrifugation, and washed three times with isopropanol, then dried in vacuum. The obtained nanocrystals could be dispersed in water to form a stable and clear colloidal solution.

### Preparation of Fe_3_O_4_ colloidal solution

A mixture of 20 mL of FeCl_3_·6H_2_O (0.1 M) and 20 mL of the MPA ligand (0.1 M) was placed in a 100 mL flask and the pH was adjusted to 11 with 2 M NaOH. The obtained solution was labelled B. The reaction mixture was then heated to reflux for 2 h and then cooled down to room temperature. A black powder was recovered by centrifugation after adding isopropanol.

### Synthesis of bifunctional core/shell/shell Mn:ZnS/ZnS/Fe_3_O_4_ nanocrystals

The bifunctional Mn:ZnS/ZnS/Fe_3_O_4_ QDs were prepared from the Mn:ZnS/ZnS colloidal solution A. 5.5 mL of solution A was added to 94.5 mL of ultrapure water and the pH was adjusted to 11 using 2 M NaOH. The mixture was heated to 100 °C and a specific amount of solution B (1, 1.5, 2 or 3 mL) was injected dropwise, resulting in the formation of Fe_3_O_4_ magnetic layers of different thicknesses covering the Mn:ZnS/ZnS cores. The samples obtained were labelled Mn:ZnS/ZnS/Fe_3_O_4_ (1), (1.5), (2) and (3), respectively. After cooling to room temperature and addition of isopropanol, the Mn:ZnS/ZnS/Fe_3_O_4_ particles were collected by centrifugation. The obtained nanocrystals could be dispersed in water to form stable magnetic and fluorescent colloidal dispersions.

### Experimental techniques

The X-ray powder diffraction data were collected from an X'Pert MPD diffractometer (Panalytical AXS) with a goniometer radius of 240 mm and using Cu Kα radiation (λ = 0.15418 nm). The average particle size was calculated from the line broadening using the Debye–Scherrer formula, *D* = *K*λ/β·cos(θ), where *D* is the average crystallite size, *K* is the shape factor taken as 0.9, λ is the X-ray wavelength, β is the full width at half maximum of the Bragg reflection in radians, and θ is the diffraction angle.

The transmission electron microscopy images were taken by placing a drop of the particles dispersed in water onto a carbon film-supported copper grid, and the size and the shape of the particles were determined using a Philips CM20 instrument operating at 200 kV.

The DLS measurements were performed on a ZEN 3600 Zetasizer Nano ZS. The nanocrystals were dispersed in water by sonication and transferred to quartz cuvettes using a syringe.

The absorption spectra were recorded on a Thermo Scientific, Evolution 220 UV–vis spectrophotometer. The photoluminescence spectra were recorded on a Horiba, Fluoromax-4, Jobin Yvon spectrofluorimeter. The QY values were determined from the following equation:

[1]
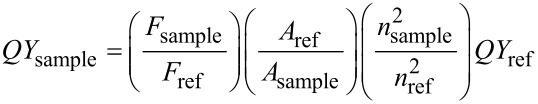


where *F*, *A* and *n* are the measured fluorescence (area under the emission peak), absorbance at the excitation wavelength and the refractive index of the solvent, respectively. The PL spectra were spectrally corrected and the quantum yields were determined relative to rhodamine 6G in ethanol (QY = 94%).

The magnetic measurements were carried out using the physical properties measurement system (PPMS) from Quantum Design. The hysteresis loops were recorded at 2 K and 300 K in the range −9 T to +9 T. The thermal variation of the magnetization was studied using zero-field-cooled (ZFC) and field-cooled (FC) procedures under an applied magnetic field of 500 Oe. For the ZFC/FC measurements, the sample was first cooled without a field from room temperature to 2 K. Thereafter, a 500 Oe magnetic field was applied and the magnetic moment was recorded upon increasing temperature to obtain the ZFC curve. For the FC curve, the sample was cooled from 300 K under a field of 500 Oe and the magnetic moment was recorded upon decreasing temperature.
